# Integrated high-throughput analysis identifies super enhancers in metastatic castration-resistant prostate cancer

**DOI:** 10.3389/fphar.2023.1191129

**Published:** 2023-05-24

**Authors:** Jie Zeng, Jiahong Chen, Maozhang Li, Chuanfan Zhong, Zezhen Liu, Yan Wang, Yuejiao Li, Funeng Jiang, Shumin Fang, Weide Zhong

**Affiliations:** ^1^ Department of Urology, The Second Affiliated Hospital, School of Medicine, South China University of Technology, Guangzhou, Guangdong, China; ^2^ Department of Urology, Huizhou Municipal Central Hospital, Huizhou, Guangdong, China; ^3^ Zhujiang Hospital, Southern Medical University, Guangzhou, Guangdong, China; ^4^ Department of Urology, Minimally Invasive Surgery Center, The First Affiliated Hospital of Guangzhou Medical University, and Guangdong Key Laboratory of Urology, Guangzhou, Guangdong, China

**Keywords:** prostate cancer, super enhancer, CUT&Tag, mCRPC, transcription factor

## Abstract

**Background:** Metastatic castration-resistant prostate cancer (mCRPC) is a highly aggressive stage of prostate cancer, and non-mutational epigenetic reprogramming plays a critical role in its progression. Super enhancers (SE), epigenetic elements, are involved in multiple tumor-promoting signaling pathways. However, the SE-mediated mechanism in mCRPC remains unclear.

**Methods:** SE-associated genes and transcription factors were identified from a cell line (C4-2B) of mCRPC by the CUT&Tag assay. Differentially expressed genes (DEGs) between mCRPC and primary prostate cancer (PCa) samples in the GSE35988 dataset were identified. What’s more, a recurrence risk prediction model was constructed based on the overlapping genes (termed SE-associated DEGs). To confirm the key SE-associated DEGs, BET inhibitor JQ1 was applied to cells to block SE-mediated transcription. Finally, single-cell analysis was performed to visualize cell subpopulations expressing the key SE-associated DEGs.

**Results:** Nine human TFs, 867 SE-associated genes and 5417 DEGs were identified. 142 overlapping SE-associated DEGs showed excellent performance in recurrence prediction. Time-dependent receiver operating characteristic (ROC) curve analysis showed strong predictive power at 1 year (0.80), 3 years (0.85), and 5 years (0.88). The efficacy of his performance has also been validated in external datasets. In addition, FKBP5 activity was significantly inhibited by JQ1.

**Conclusion:** We present a landscape of SE and their associated genes in mCPRC, and discuss the potential clinical implications of these findings in terms of their translation to the clinic.

## Introduction

Prostate cancer (PCa) is a prevalent malignant tumor affecting the male genitourinary system (Siegel, et al., 2021). According to the World Health Organization (WHO) 2020 GLOBOCAN statistics, it ranks second in incidence among malignancies in men globally, after lung cancer, and has the fifth highest mortality rate among all malignancies in men (Sung, et al., 2021). Progression from local PCa to castration-resistant prostate cancer (CRPC) is inevitable (Davies, et al., 2019). Approximately 35% of patients with early-stage localized cancer and 50% of locally advanced prostate cancer have recurrence and metastasis (Djavan, et al., 2003). And the 5-year survival rate of metastatic prostate cancer is 28% (Nandana and Chung, 2014).

Epigenetic programming has emerged as a critical step in the activation and maintenance of aberrant transcriptional programs in CRPC pathogenesis (Cimadamore, et al., 2017; Yegnasubramanian, et al., 2019; Sugiura, et al., 2021). Recent studies have shown that hypermethylation of androgen receptor (AR) leads to loss of AR expression in CRPC patients (up to 30%) (Suzuki, et al., 2003; Chmelar, et al., 2007), driving CRPC progression. Epigenetic regulatory heterogeneity could lead to intratumoral phenotypic plasticity (Ateeq, et al., 2016). Phenotypic plasticity increases the probability of tumor cells successfully migrating to and surviving in different metastatic environments (Klein, 2013). In addition, nonmutational epigenetic reprogramming, an emerging feature, has been added to the list of hallmarks of cancer (Hanahan, 2022).

Super enhancers (SEs) are important elements of epigenetic regulation (Hah, et al., 2015; Thandapani, 2019), the concept of which was first proposed by Professor Young R.A. (Whitehead Institute for Biomedical Research) in 2013. SEs are a large cluster of active transcriptional enhancers spanning a long-range region of genomic DNA. SE binding sites are occupied by high-density transcription factors (TFs), coactivators (mediators), and histone modification marks (Ing-Simmons, et al., 2015; Sengupta and George, 2017). Compared to typical enhancers (TEs), which only recruit one TF, SEs recruit multiple TFs to one site and drive stronger transcriptional activation. Therefore, SEs participate in multiple signaling pathways and facilitate tumor-promoting gene changes (Hnisz, et al., 2015). SEs are essential for maintaining the stemness of embryonic stem cells (Whyte, et al., 2013) and maintaining tumor characteristics by facilitating special gene expression patterns (Hnisz, et al., 2013; Thandapani, 2019).

Specifically, SEs facilitate the dysregulation of transcriptional programs mediated by BRD4 (Urbanucci and Mills, 2018), CDK7, ERG, and other factors in PCa cells. BRD4, a member of the bromodomain and extraterminal domains (BETs) family (Chen et al., 2020), is a critical SE-related protein in PCa (Donati, et al., 2018; Shafran, et al., 2019). It acts as an epigenetic “reader” that binds to specific acetylated lysine residues on histone tails, facilitating the assembly of transcription complexes. BRD4 exhibits dense binding activity in SE and drives cell-identical gene expression (Lee, et al., 2017). In particular, BRD4 physically interacts with the N-terminal domain of AR, driving AR-mediated gene transcription. AR signaling remains the most common resistance mechanism in most CRPC patients (Dai, et al., 2017; Aurilio, et al., 2020). Moreover, BET inhibitors could resensitize drug-resistant CRPCs to enzalutamide (Shah, et al., 2017). The above evidence suggests that SE may be closely related to mCRPC.

To reveal the epigenetic dysregulation mechanism of mCRPC, SEs and TFs were first screened from C4-2B cells by CUT&Tag. We present a landscape of SE and their associated genes in mCPRC.

## Materials and methods

### Cell culture

The human PCa cell line C4-2B, a subline of human PCa LNCaP-derived C4-2 cells, was purchased from the BeNa Culture Collection (BNCC) (BNCC341733). Cells were cultured at 37°C in an atmosphere humidified with 20% O2 and 5% CO_2_ and were cultivated in RPMI-1640 medium (MA0215, Meilunbio) containing 10% fetal bovine serum (A3160802, Gibco) and 1% (100 μg/mL) penicillin/streptomycin (15140-122, Gibco).

### Cell line treatment conditions

To verify the hub SE-associated DEGs, C4-2B cells were treated with 500 nM and 2 μM JQ1 (CAS No.:1268524-70-4, MedChemExpree) for 24 h. Equal volumes of the carrier (DMSO) were used as control.

### High-throughput CUT&Tag

The Cleavage Under Targets and Tag mentation (CUT&Tag) assay was performed as previously described (Kaya-Okur, et al., 2019). Briefly, 1 × 10^5^ C4-2B cells were carefully washed twice with wash buffer (20 mM HEPES pH 7.5; 150 mM NaCl; 0.5 mM spermidine; 1× protease inhibitor cocktail). Ten microliters of concanavalin A-coated magnetic beads (Bangs Laboratories) were added to each sample and incubated at RT for 10 min. The unbound supernatant was removed, and bead-bound cells were resuspended in dig wash buffer (20 mM HEPES pH 7.5; 150 mM NaCl; 0.5 mM spermidine; 1× protease inhibitor cocktail; 0.05% digitonin; 2 mM EDTA) and a 1:50 dilution of primary antibody (ab4729 for H3K27ac, Abcam; ab8895 for H3K4me1, Abcam). Then, the cells were incubated overnight at 4°C on a rotating platform. The primary antibody was removed using a magnet stand. Secondary antibody (goat monoclonal: Millipore AP132) was diluted 1:100 in digitonin wash buffer, and the cells were incubated at room temperature for 1 h. The cells were washed 3 times with a magnet stand in digitonin wash buffer. A 1:100 dilution of the pA-Tn5 adapter complex was prepared in Dig-med Buffer (0.01% digitonin; 20 mM HEPES pH 7.5; 300 mM NaCl; 0.5 mM spermidine; 1× protease inhibitor cocktail) and incubated with cells at room temperature for 1 h. The cells were washed 3× for 5 min in 1 mL Dig-med buffer, resuspended in tagmentation buffer (10 mM MgCl_2_ in Dig-med Buffer) and incubated at 37°C for 1 h. DNA was purified using phenol‒chloroform-isoamyl alcohol extraction and ethanol precipitation. For amplification of the libraries, 21 μL DNA was mixed with 2 μL of a universal i5 primer and a uniquely barcoded i7 primer. A volume of 25 μL NEBNext HiFi 2× PCR Master mix was added, and the sample was mixed. The sample was placed in a thermocycler with a heated lid, and the following cycling conditions were applied: 72°C for 5 min (gap filling); 98°C for 30 s; 14 cycles of 98°C for 10 s and 63°C for 30 s; final extension at 72°C for 1 min and holding at 8°C. The library clean-up was performed with XP beads (Beckman Counter).

### Identification of predicted SEs and TFs

H3 lysine 4 monomethylation (H3K4me1) and H3K27 acetylation (H3K27ac) (Creyghton, et al., 2010), both active histones, are associated with active enhancers and the presence of actively transcribing Pol II (McVicker, et al., 2013). Therefore, antibodies against active histones were used to bind target chromatin proteins between nucleosomes in the genome. Then, target peaks were detected in purified DNA by CUT&Tag (Kaya-Okur, et al., 2019). Active enhancers were those enriched in both H3K4me1 and H3K27ac (Li, et al., 2020). H3K4me1 can mark active or poised enhancers. H3K27ac distinguishes active enhancers from inactive/poised enhancer elements. Thus, the H3K4me1 peaks file identified by MACS2 and the H3K27ac BAM file were used as input to the algorithm firstly. Then, sort the enhancer according to the signal value of H3K27ac by SEs software (ROSE). The regions of SE or TE units and the 200 bp extending on both sides were used as input regions. Then, hypergeometric optimization of motif enrichment (HOMER) was used to predict enriched motifs. Detailed information about TFs was obtained from the Human TFs website (http://humantfs.ccbr.utoronto.ca/) (Lambert, et al., 2018). Information on over 1,600 human TFs has been deposited on this website.

### Data acquisition

GSE35988 (Grasso, et al., 2012), containing mCRPC patients, was obtained from Gene Expression Omnibus (GEO, http://www.ncbi.nlm.nih.gov/geo). Then, DEGs between mCRPC (*n* = 27) and localized PCa (*n* = 49) tissue were compared via the online tool PCaDB (http://bioinfo.jialab-ucr.org/PCaDB/). The analysis method was limma, and the thresholds were |fold change| > 2 and *p*-value <0.01.

The training cohort (Taylor) and the validation cohorts (TCGA-PRAD, DKZF, GSE54460, CancerMap, Cambridge, Belfast, CPC-Gene, and Stockholm) of SE-associtated DEGs prognostic model were downloaded from PCaDB.

Single-cell RNA-seq of mCRPC tissue was performed by the Dana-Farber/Harvard Cancer Center Institutional Review Board with ethics approval and informed consent (He, et al., 2021).

Gene expression values of C4-2B, PC-3, LNCaP under JQ1 treatment were extracted from GSE98069 (Coleman, et al., 2019) using the edgeR package (Shen, et al., 2021).

### Functional enrichment analysis

SE-associated DEGs are the intersection of SE-associated genes and DEGs of mCRPC. Enrichment analyses of SE-associated genes, DEGs and SE-associated DEGs were performed based on various online databases. Hallmark enrichment analysis based on The Molecular Signatures Database (MSigDB) was performed using gene set enrichment analysis (GSEA). In addition, genes were subjected to Kyoto Encyclopedia of Genes and Genomes (KEGG) and Gene Ontology (GO) enrichment analysis. |Fold change|>1 and *p*-value < 0.05 were considered to indicate statistical significance.

### Ratio model training

For SE-associtated DEGs prognostic model training, 94 upregulated SE-associated DEGs and 48 downregulated SE-associated DEGs were used as candidates; CoxRidge, a package for fitting Cox models with penalized ridge-type partial likelihood, was used as the method.

$$SE-associated\;DEGs\;score=\sum_{i=1}^{N}\left(Coefficienti\times Expression\;level\;of\;mRNAi\right)$$

Where “N” (N = 13) represents the total number of the SE-associated DEGs in the training model, “Coefficient_i_” denotes a specific mRNA’s coefficient of SE-associated genes, and “Expression level of mRNAi” refers to the relative expression level of a certain mRNA.

Given the median scores in Taylor dataset, the low risk group and high risk group were defined. The Kaplan-Meier (KM) survival analysis depicted the BCR-free survival probability curves between the low risk group and high risk group. ROC curves of 1-, 3-, and 5-year evaluate the predictive power of SE-associated DEGs score.

Subsequently, 8 independent validation cohorts (TCGA-PRAD, DKZF, GSE54460, CancerMap, Cambridge, Belfast, CPC-Gene, and Stockholm) were used for depicted the BCR-free survival probability, which are the classic PCa datasets currently retrievable. Forest plots (http://vip.sangerbox.com/home.html) were used for visualization.

### RNA isolation and RT‒qPCR

Total RNA was extracted from C4-2B cells using the RNA Quick Purification Kit (RN001; esunbio) according to the manufacturer’s instructions. The RNA concentration was measured using a DS -11+ Spectrophotometer (DeNovix, United States). cDNA was synthesized from the extracted RNA by reverse transcription using HiScript II Q RT SuperMix for qPCR (+gDNA wiper) (R223; Vazyme Biotech) in the Veriti™ 96-Well Fast Thermal Cycler (catalog number: 4375305, Thermo Fisher Scientific). RT‒qPCR was performed using ChamQ Universal SYBR qPCR Master Mix (Q711-02; Vazyme Biotech) and a QuantStudio™ 1 Real-Time PCR System (catalog number: A40427, Thermo Fisher Scientific). Primers were chemically synthesized by Tsingke Biotechnology Co., Ltd. (Guangzhou, China).

Primer sequences for amplification are listed in the supplemental information (Supplementary Table S9).

### Interaction network analyses

TFs were predicted by HOMER’s findMotifsGenome.pl tool based on the C4-2B CUT&Tag peak file. The interaction network between TFs and SE-associated DEGs was determined by utilizing the STRING (http://string-db.org) online database and then constructed using Cytoscape software.

## Results

### Flowchart of this study

**FIGURE 1:**
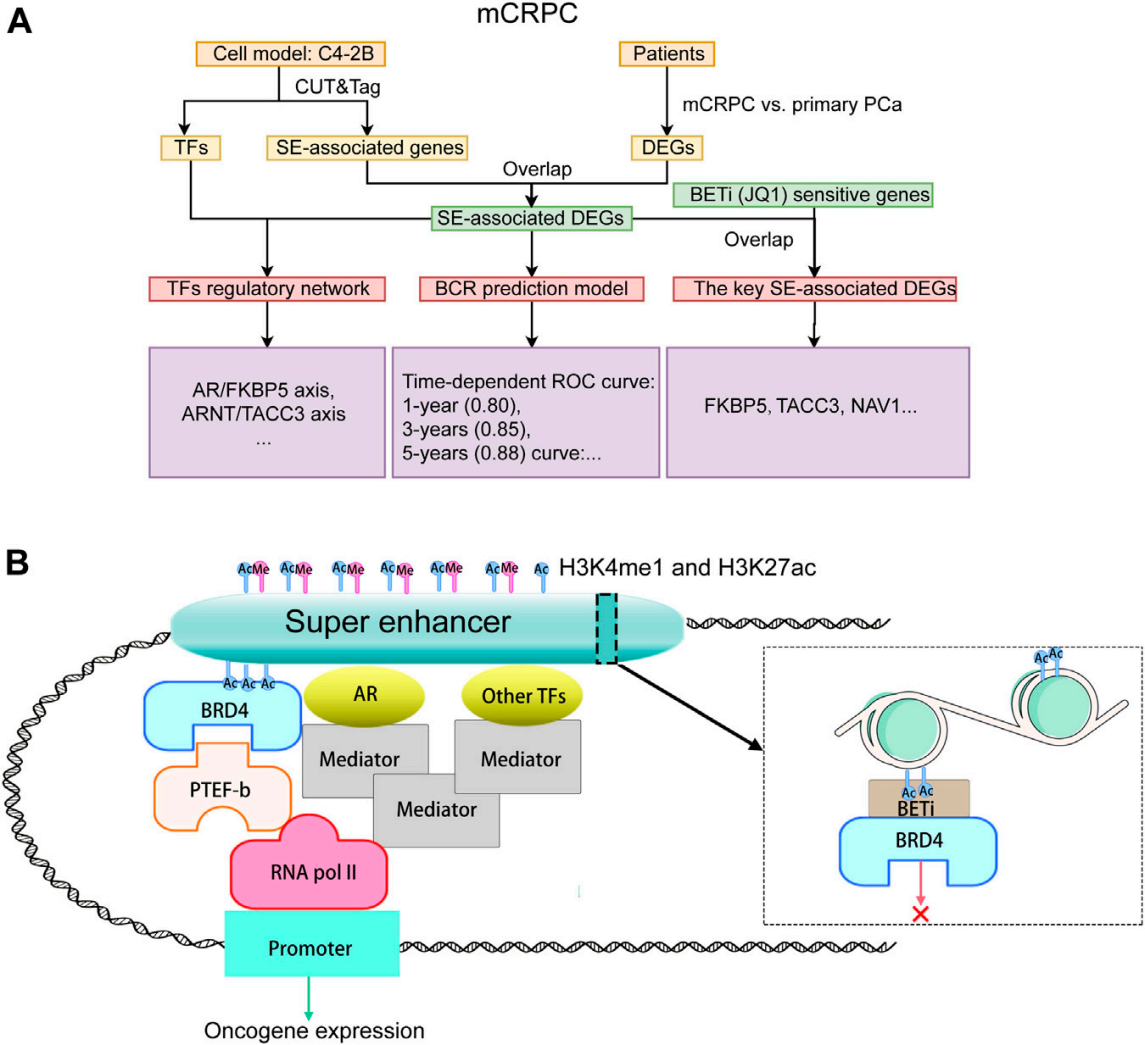
Study design and pathway diagram. **(A)** Study design. **(B)** Pathway diagram.

The study design is illustrated in Figure 1A. To reveal the epigenetic dysregulation mechanism of mCRPC, super-enhancer (SE) associated genes and transcription factors were first screened from C4-2B cellsusing CUT&Tag assay. The differentially expressed genes related to mCRPC were identified from the GSE35988 dataset, and the SE-associated DEGs were obtained by taking the intersection of SE-associated genes and DEGs. Subsequently, a recurrence prediction model was constructed based on SE-associated DEGs, and a SE-mediated TF regulatory network was built using TFs and SE-associated DEGs. JQ1, a BET inhibit BCR or, was added to block SE-mediated TF transcription and identify key SE-associated DEGs, which were found to include FKBP5 and TACC3 genes. The hypothesis diagram depicted in Figure 1B suggests that H3K4me1 and H3K27ac histone marks can recruit active SEs, which can bind multiple TFs to drive stronger transcriptional activation.

### Identification of SE-associated genes

SEs can be found according to H3K4me1 and H3K27ac histone marks. Specifically, H3K4me1 enrichment indicates regions related to poised or less active enhancers. Enrichment of H3K27ac is a marker of active regulatory elements, including enhancers and promoters. Therefore, to identify SEs of C4-2B, a CUT&Tag assay was performed with H3K4me1 and H3K27ac. H3K4me1 can mark active enhancers as well as those in a poised or predetermined state. And H3K27ac distinguishes active enhancers from inactive/poised enhancer elements containing H3K4me1 alone (Creyghton, et al., 2010).

**FIGURE 2:**
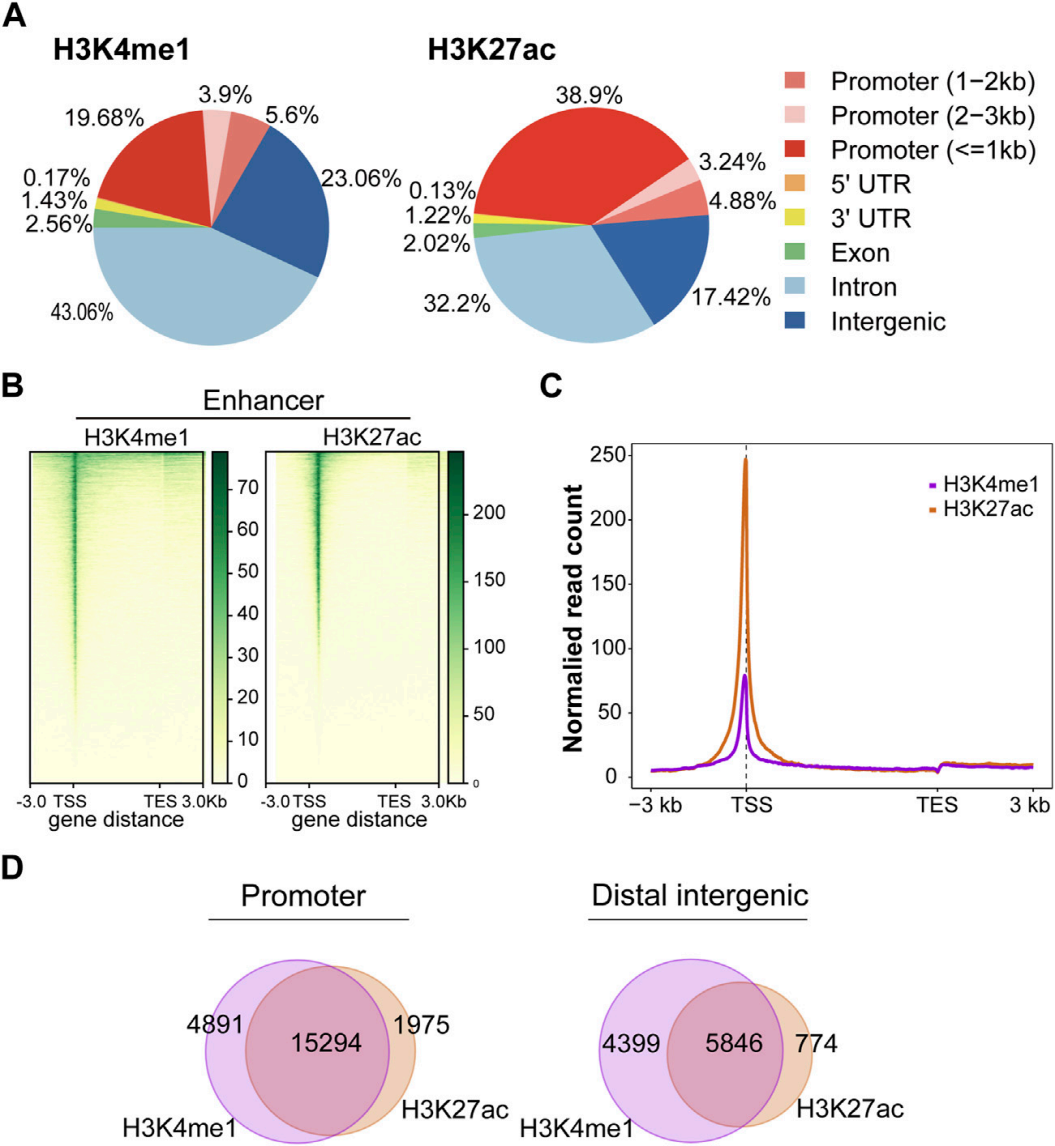
Active histone mark distributions in C4-2B cells. **(A)** The distribution of genomic regions modified by H3K4me1 and H3K27ac, which were classified into six region types (promoter, 5′UTR, 3′UTR, exon, intron, and intergenic). **(B)** Heatmap of active histone modifications detected in C4-2B cells. **(C)** Density profiles of H3K4me1 (purple) and H3K27ac (orange) at C4-2B. Profiles included 3 kb upstream of TSS and 3 kb downstream of TES. **(D)** The overlapping region between H3K4me1 and H3K27ac in promoters and distal intergenic regions.

In this study, we firstly evaluated the epigenetic landscape based on two active histone marks (H3K4me1 and H3K27ac). Annotations of the peaks showed that H3K4me1 modification was mainly found in intron (43.06%) and distal intergenic (23.06%) regions. In contrast, H3K27ac modification was mainly found in promoter (47.02%) and intron (32.2%) regions (Figure 2A). The H3K4me1 and H3K27ac marks showed the same profile surrounding the transcription start site (TSS) (Figures 2B, C). H3K4me1 and H3K27ac histone marks identify regions that likely contain enhancers. Importantly, SEs could be found in regions with H3K4me1 and H3K27ac histone marks. Moreover, the peaks of H3K4me1 and H3K27ac were highly colocalized in the promoter and distal intergenic regions (Figure 2D), suggesting that H3K4me1 and H3K27ac histone marks were also highly enriched in the promoter and distal intergenic regions.

**FIGURE 3:**
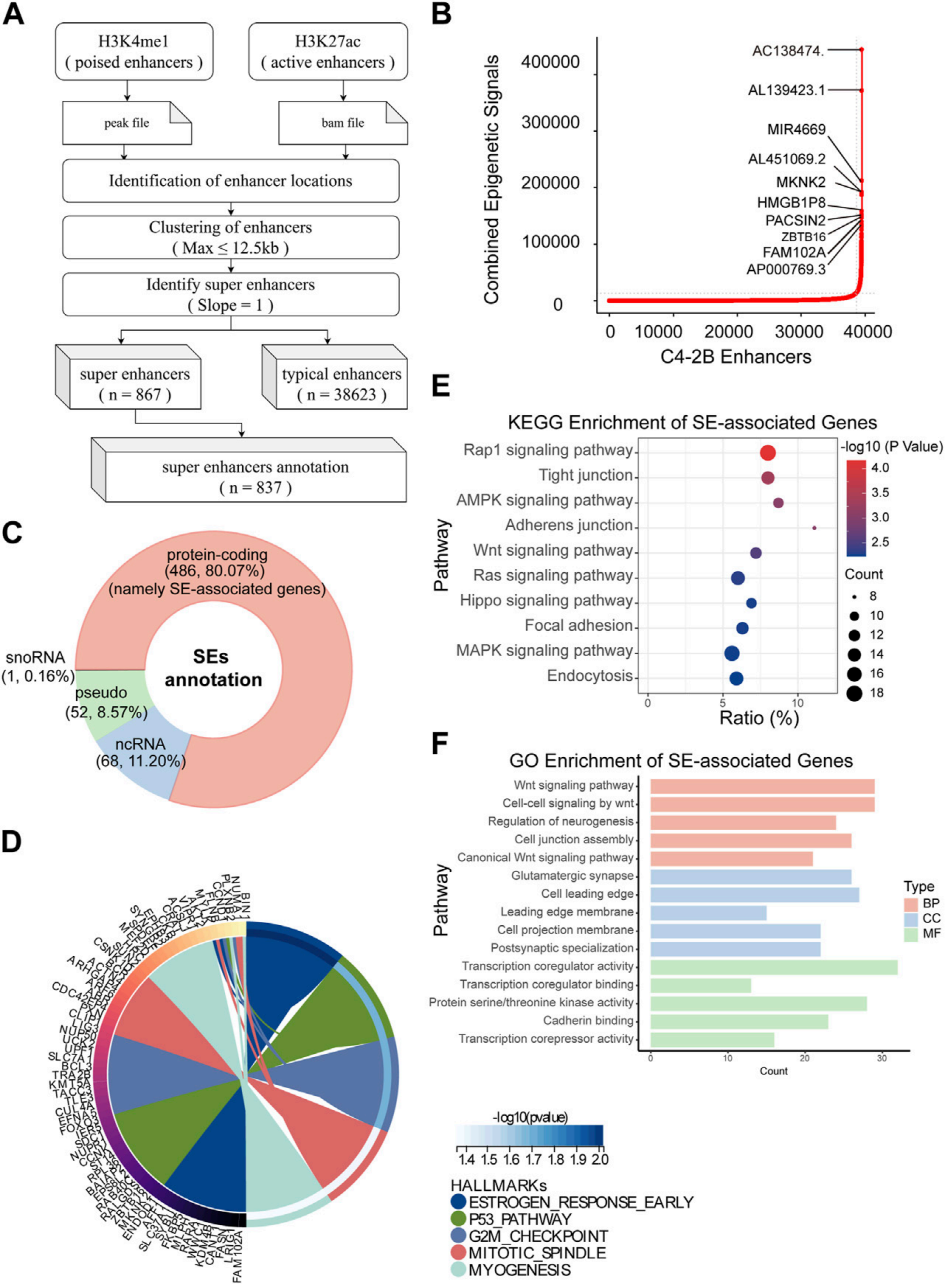
Identification of SEs in C4-2B cells. **(A)** The flow of SE identification. **(B)** Ranking of enhancers using the ROSE algorithm. **(C)** Classification of SE-related genes; protein-coding RNAs, namely, SE-associated genes, were the focus. **(D–F)** Hallmark, KEGG and GO enrichment analyses of SE-associated genes in C4-2B cells.

Immediately afterward, we identified SEs and their associated genes. H3K4me1 is used to find out poised enhancer. Then, sort the enhancer according to the signal value of H3K27ac by SE software (ROSE) (Figure 3A). According to the rank ordering of ROSE, 867 activated SEs were identified, and the cutoff value was 13537.2441 (Figure 3B). These SEs could be annotated to 867 genes (Supplementary Table S1). Among them, there were 486 protein-coding RNAs, 68 ncRNAs, 50 pseudogenes and 1 snoRNA. In the present study, only protein-coding RNA was selected for further investigation. Therefore, we defined 486 SE-related protein-coding RNAs as SE-associated genes (Figure 3C). Hallmark enrichment analysis showed that C4-2B may be greatly influenced by the early estrogen response, P53 signaling pathway, and G2/M checkpoint (Figure 3D). KEGG analysis revealed that the SE-associated genes are involved in the Rap1 signaling pathway, tight junctions, the AMPK signaling pathway, and adherens junctions (Figure 3E). GO enrichment analysis confirmed the perturbation of Wnt signaling pathways (Figure 3F).

Overall, we not only screened 486 SE-associated genes related to mCRPC but also proposed a possible mechanism connecting mCRPC and the Rap1 signaling pathway, tight junctions, the AMPK signaling pathway, or adherens junctions.

### Analysis of the differential expression pattern of mCRPC

**FIGURE 4:**
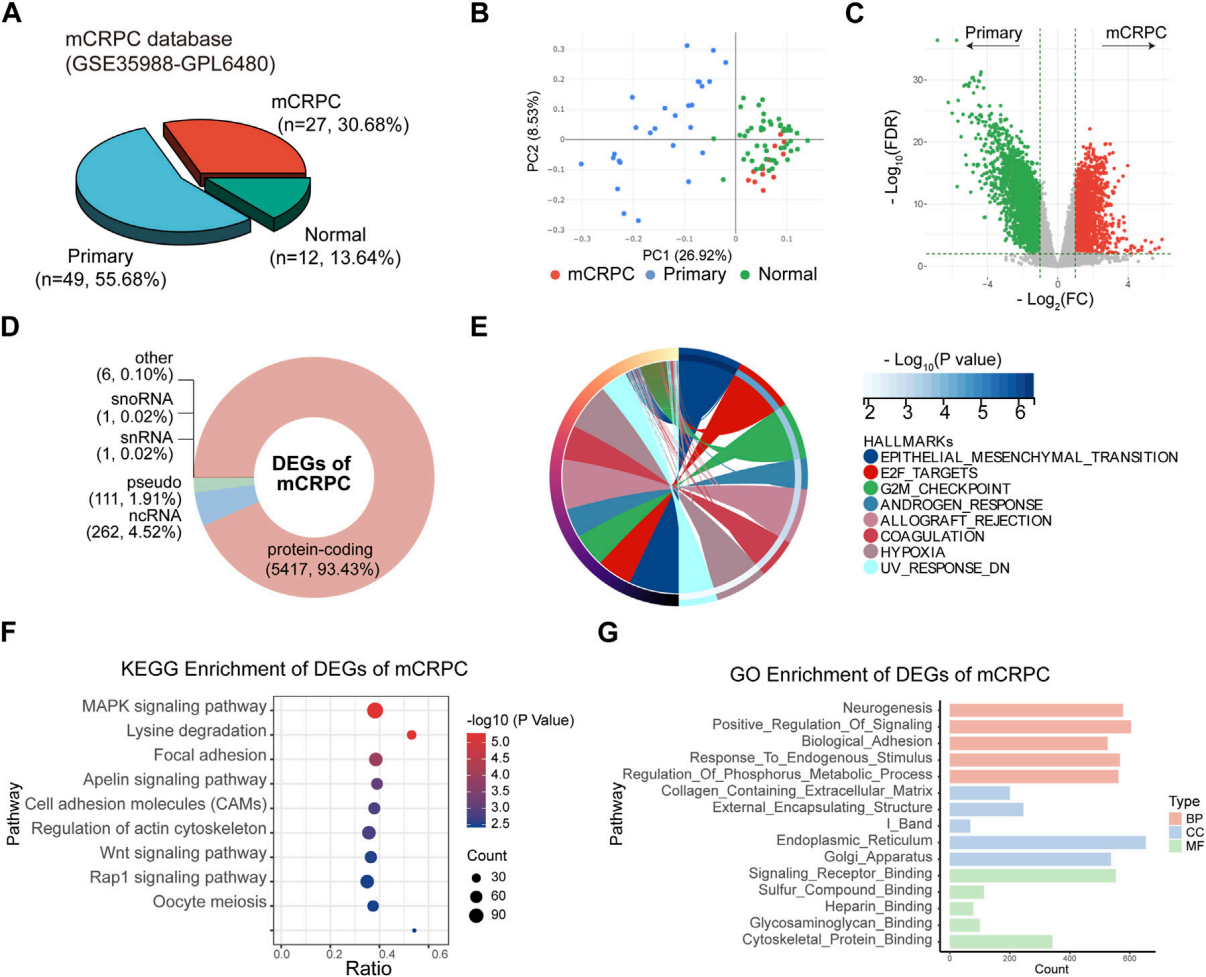
Identification of DEGs between mCRPC and primary PCa patients in GSE35988. **(A)** Composition of sample types in GSE35988 dataset. **(B)** Principal component analysis (PCA) of GSE35988 dataset. **(C)** Volcano plot of DEGs between mCRPC patients and primary PCa patients. (*p* < 0.01, |fold change| > 2, limma methods). **(D)** Classification of DEGs. **(E–G)** Hallmark, KEGG and GO enrichment analyses of DEGs of mCRPC.

The GSE35988 dataset contains information on 49 localized PCa patients and 27 mCRPC patients (Figure 4A) and was used for differential expression pattern analysis of mCRPC. First, we observed that localized PCa patients and mCRPC patients could be divided into two groups according to principal component analysis (PCA) (Figure 4B). Then, we identified 5,798 statistically significant DEGs between the two groups (Figure 4C; Supplementary Table S2). These DEGs of mCRPC could be divided into 5,417 protein-coding RNAs, 262 ncRNA, 111 pseudogenes, and 1 snoRNA. and 1 snRNA (Figure 4D). Subsequently, 5,417 protein-coding RNAs were used for enrichment analysis. In the hallmark enrichment analysis, DEGs were found to be enriched in epithelial-mesenchymal transformation, E2F targets, the G2/M checkpoint, and the androgen response (Figure 4E). KEGG enrichment analysis showed that DEGs were involved in the MAPK-type pathway, focal adhesion and the Rap1 signaling pathway (Figure 4F). Moreover, GO enrichment analysis demonstrated that Neurogenesis was the main enriched term for DEGs in mCRPC (Figure 4G).

### Application of SE-associated DEGs to predict biochemical recurrence (BCR) in PCa patients

**FIGURE 5:**
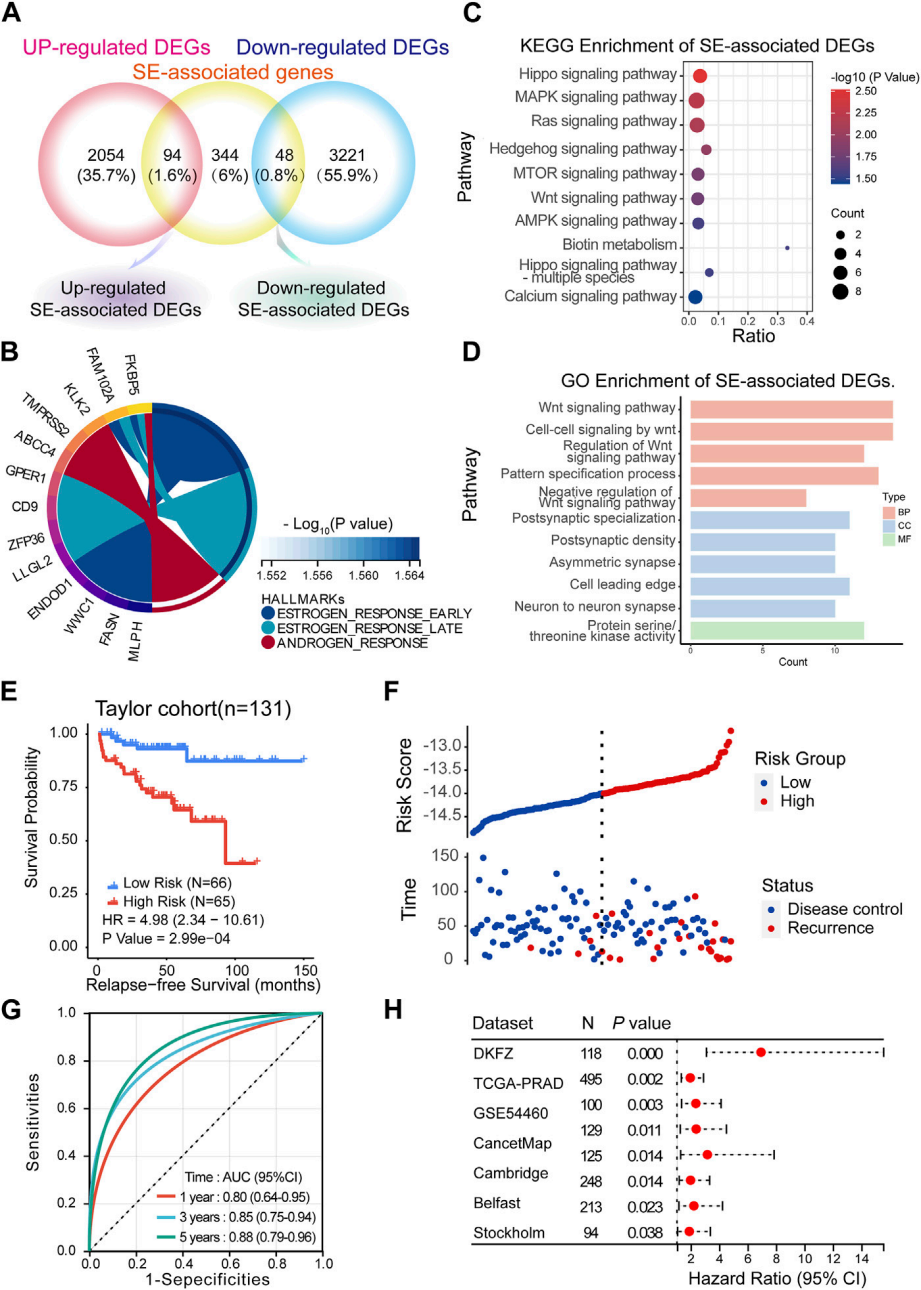
Predicting BCR based on the SE-associated DEGs model. **(A)** Screening of SE-associated DEGs. **(B–D)** Hallmark, KEGG and GO enrichment analyses of SE-associated DEGs. **(E,F)** Recurrence-free survival (RFS) in the training cohort (Taylor) (Method: CoxRidge). **(G)** ROC curves test the predictive value of the risk score in the training cohort. **(H)** Forest plot analysis of RFS in the validation cohort.

SEs have been considered important epigenetic regulators. Therefore, we aimed to investigate the clinical significance of SEs in mCRPC. First, we took the intersection of 486 specific SE-associated genes, 2,418 upregulated DEGs and 3,269 downregulated DEGs. We obtained 94 upregulated SE-associated DEGs and 48 downregulated SE-associated DEGs (Figure 5A; Supplementary Table S3). These SE-associated DEGs were presumed to be involved in the progression. Thus, we performed hallmark, KEGG, and GO enrichment analyses of SE-associated DEGs. Hallmark enrichment analysis showed that estrogen response signaling and androgen response signaling were the most prominent signaling pathways (Figure 5B). KEGG enrichment analyses showed that the Hippo signaling pathway, MAPK signaling pathway, and Ras signaling pathway may be vital (Figure 5C). The GO enrichment analysis supported the Wnt signaling pathway result (Figure 5D).

BCR, which is characterized by an increase in prostate-specific antigen (PSA) after completed surgery (Moul, 2000) or radiation (Roach et al., 2006), was used to reflect the treatment effect. Here, we established a BCR prediction model based on SE-associated DEGs. First, the above SE-associated DEGs were selected for training in the Taylor dataset using the CoxRidge. Kaplan–Meier survival analysis of biochemical recurrence-free survival (BRFS) showed a significantly worse prognosis in the high-risk group, implying that the high-risk group was likely to relapse earlier than the low-risk group in the Taylor cohort (HR = 4.98) (Figures 5E, F; Supplementary Tables S4, S5). Time-dependent ROC curve analysis revealed that the BCR model performed well in predicting outcomes at 1 year (0.80), 3 years (0.85), and 5 years (0.88) (Figure 5F). Consistently, the model of SE-associtated DEGs also predicted BRFS well in multiple established validation cohorts (Figure 5G, Supplementary Table S6), such as the TCGA-PRAD, DKZF, GSE54460, CancerMap, Cambridge, Belfast, CPC-Gene, and Stockholm cohorts.

### TF regulatory network of mCRPC

Together, SEs, TFs, and multiple genes form a transcriptional regulatory loop. Notably, TFs in particular play a crucial role in SE-mediated transcriptional regulation. To explore the mechanism of epigenetic dysregulation of mCRPC, we predicted TFs to establish a TF regulatory network, which consisted of three layers of elements of regulation: SEs, TFs and SE-associated DEGs.

**FIGURE 6:**
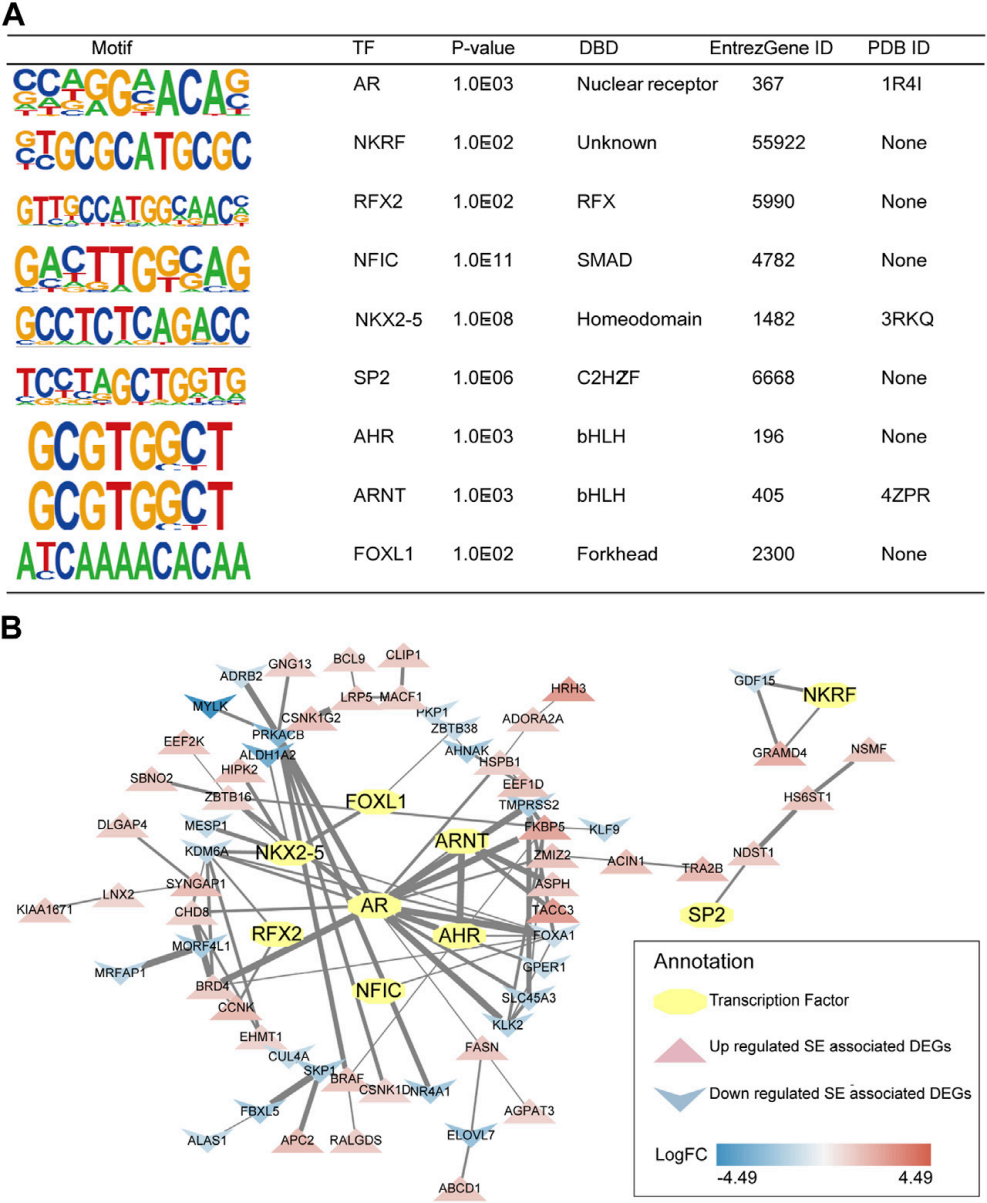
Predicted TFs and their interaction with SE-associated DEGs. **(A)** Detailed information of selected human TFs. **(B)** TFs regulatory network of mCRPC.

First, TFs of mCRPC were predicted by HOMER based on the DNA-binding motifs of C4-2B. HOMER motif discovery revealed 14 known motifs and 36 *de novo* motifs as the highest-scoring motifs (*p* < 0.01). Analysis of these known motifs revealed 3 human TFs: AR, NKRF and RFX2. Moreover, *de novo* motif analysis identified 6 probable human TFs, including NFIC, NKX2-5, SP2, AHR, ARNT and FOXL1. According to DNA-binding domain (DAB) classification, AR is a TF of nuclear receptors; RFX2 is a TF of regulatory factor binding to the X-box (RFX); NFIC is a TF of SMAD; NKX2-5 is a TF of homeodomain genes; SP2 is a TF of Cis2-His2 zinc finger (C2H2-ZF); AHR and ARNT are basic helix-loop-helix (bHLH) TFs; and FOXL1 is a forkhead box (FOX) TF (Figure 6A).

Subsequently, 3 human TFs revealed by known motif analysis and 6 human TFs identified by *de novo* motif analysis were found to interact with SE-associated DEGs using the STRING database and visualized by Cytoscape. The TF regulatory network of mCRPC was constructed from TFs and their potential regulatory SE-associated DEGs (Figure 6B).

Consistent with a previous report (Mulholland, et al., 2011), AR was associated with FKBP5 in CRPC. FKBP5 is considered to be an androgen-inducible gene that physically interacts with AR (Zheng, et al., 2015). Moreover, the ARNT/TACC3 axis was apparent in the TF regulatory network. An association study showed that TACC3 was markedly upregulated in CRPC (Qie, et al., 2020). In addition, the ARNT/TACC3 complex is active in a hypoxic environment (Guo, et al., 2013), a signature of the tumor environment. Thus, it is reasonable to assume that progression to mCRPC may be regulated by the AR/FKBP5 and ARNT/TACC3 axis.

### Harnessing inhibitor sensitivity reveals hub genes

The bromodomain and extraterminal (BET) family, including BRD2, BRD3, and BRD4, which are partially enriched at termed SEs (Ribeiro, et al., 2012; Jin, et al., 2019), can regulate the activity of SEs and promote SE-mediated transcriptional regulatory programs (Shankar, et al., 2015). In other words, some SE-related genes could be affected by BET inhibitors. Surprisingly, JQ1, a known BET inhibitor, was reported to block the growth of PCa cells (Chen and Song, 2016; Coleman, et al., 2019). Therefore, JQ1 was used in the present study for further screening of SE-associated CRPC recurrence genes.

**FIGURE 7:**
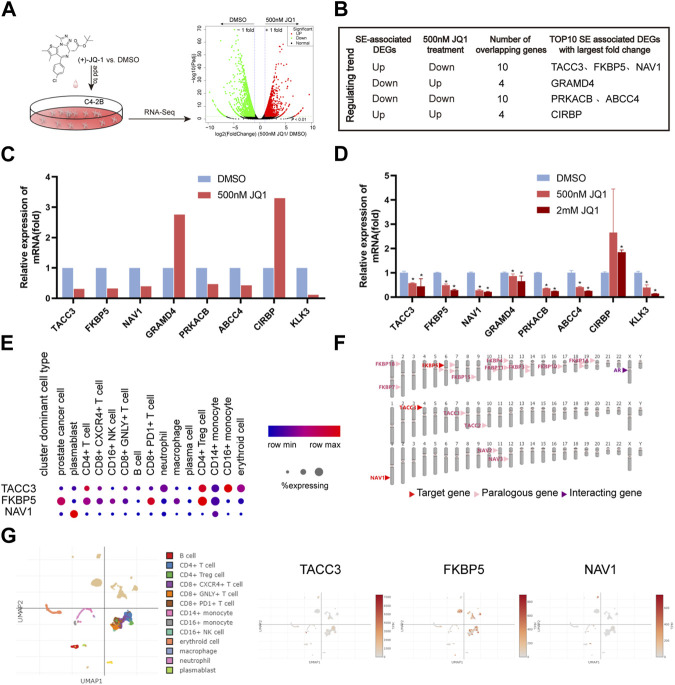
Verification of SE-associated CRPC recurrence genes. **(A)** Fast screening of BET inhibitor-sensitive SE-associated DEGs. **(B)** Selection of SE-associated CRPC recurrence genes. **(C)** Relative expression of selected genes based on GSE98069 (***p < 0.001, 500 nM JQ1 vs. DMSO). **(D)** Verification of selected genes by RT‒qPCR (***p < 0.001, 500 nM or 2 mM JQ1 vs. DMSO). **(E–G)** Single-cell analysis of TACC3, FKBP5 and NAV1.

First, the publicly available dataset GSE98069 was searched and used to rapidly detect the JQ1-sensitive genes of C4-2B. Treatment with 500 nM JQ1 for 24 h, the appropriate dose and time period, significantly reduced proliferation (Urbanucci and Mills, 2018). A total of 1423 genes were upregulated and 1680 genes were downregulated in C4-2B (Figure 7A; Supplementary Table S7). Of SE-associated CRPC recurrence genes, a total of 10 highly expressed SE-associated CRPC recurrence genes were downregulated by JQ1 treatment (Figure 7B). Among them, TACC3, FKBP5 and NAV1 had the largest fold changes and greatest significant. Next, the intersecting genes among the top 10 SE-associated genes (in terms of fold change) and JQ1-sensitive genes, which are considered to be key SE-mediated regulatory genes, were selected for PCR validation. Strikingly, KLK3, the protein-coding RNA of AR, was selected as a positive control and has been reported to be downregulated by JQ1 in PCa cells (Chen and Song, 2016) (Figure 7C; Supplementary Table S8). Our RT‒qPCR results were consistent with the RNA-Seq results of C4-2B cells treated with 500 nM JQ1. To further validate the expression changes of intersecting genes, we treated C4-2B cells with a higher dose of JQ1 (2 mM). These results indicated that even a high dose caused expression changes (Figure 7D).

To simplify, TACC3, FKBP5, and NAV1 were the most likely SE-mediated regulatory genes, and they were expressed at higher levels in mCRPC than in localized PCa. We further investigated the localization of TACC3, FKBP5, and NAV1 in single cells isolated from prostate tissue of CRPC patients. FKBP5 was obviously highly expressed in PCa cells. TACC3 was highly expressed in CD4^+^ Treg cells and CD14^+^ monocytes. NAV1 was mainly expressed in plasmablasts (Figures 7E, G). Surprisingly, we found that FKBP5 interacts closely with AR (Figure 7F). Strikingly, FKBP5 is considered to be an androgen-inducible gene that physically interacts with AR (Zheng, et al., 2015).

## Discussion

The therapeutic options are limited in the stage of mCRPC patients. SEs, epigenetic regulators, have been implicated in tumorigenesis (Cucchiara, et al., 2017; Hankey, et al., 2020). Therefore, inhibtion of SEs might be a valid strategy in the treatment of patients with refractory mCRPC.

In this study, we characterized the landscape of active histone modifications in C4-2B cells representative of mCRPC. H3K4me1 and H3K27ac are known markers of active enhancers and important indicators of enhancer activity. In particular, H3K4me1 is located in many type-specific enhancer sites. Most enhancer regions labeled with H3K4me1 are active (Calmasini, et al., 2020). H3K27ac is a candidate for distinguishing active from inactive enhancer elements (Xu, et al., 2018) A total of 867 SEs were identified in C4-2B cells by detecting both H3K4me1-and H3K27ac-enriched regions (Xu, et al., 2018). Our data showed that SE-associated genes involved in the early estrogen response and G2/M checkpoint contribute to the pathogenesis of mCRPC. Analysis of mCRPC tissues also supported this conclusion. To further prove this hypothesis, we overlapped SE-associated genes in C4-2B cells *versus* normal control cells and DEGs in mCRPC *versus* primary PCa. The overlapping genes were involved in the estrogen response, androgen response, Hippo signaling pathway, MAPK signaling pathway, and so on. These genes are closely related to mCRPC.

Local therapies, including radical prostatectomy or primary definitive radiotherapy, are the initial treatments for primary PCa. However, many patients will eventually reach the mCRPC stage (Cornford, et al., 2021). BCR reflects the effects of treatment. Therefore, SE-associated DEGs, namely, SE-associated mCRPC recurrence genes, were used to construct the BCR prediction model. This model displayed a significantly excellent predictive power for mCRPC recurrence at the 1-year, 3-year and 5-year follow-up. This implies that mCRPC patients with high predictive scores might benefit from BET inhibitors.

SEs activate transcription by recruiting TFs. Therefore, we further sought TFs of mCRPC and then established a TF regulatory network. Reliable studies have summarized 1,639 known or probable human TFs (Lambert, et al., 2018). Fortunately, 9 human TFs were explored in C4-2B cell lines. Undoubtedly, it has been widely assumed that AR signaling is related to CRPC biology (Watson, et al., 2015). The AR gene body and/or the enhancer are amplified in 81% of mCRPC cases (Quigley, et al., 2018; Zhao, et al., 2020). NF-κB repressing factor (NKRF), an inhibitor of NF-KB-mediated gene transcription, has been associated with tumor invasion, migration, and progression (Hsu, et al., 2012; Xu, et al., 2022). Regulatory factor X2 (RFX2) has been identified as the major master TF in regulating the angiogenesis signature in renal carcinoma (Zheng, et al., 2021). Furthermore, the RFX2/RFX3 complex could only be detected in the nuclear extract of FGF-1B-positive cells; it directly binds the 18-bp cis-element (−484 to −467) and contributes to the regulation of the fibroblast growth factor 1 (FGF1) promoter, which has been shown to regulate cell proliferation and cell division (Hsu, et al., 2012).

Acetylated chromatin, particularly in SE regions, is associated with bromodomain and extraterminal (BET) proteins to facilitate transcriptional activation (Li, et al., 2020). BET inhibitors disrupt the interaction of bromodomains with acetylated histones and result in the loss of enhancer-promoter long-range interactions. Several BET inhibitors are currently being investigated in phase I/II in mCRPC patients, including ZEN003694 (NCT02711956, NCT02705469), GS-5829(NCT02607228), and OTX015/MK-8628 (NCT02259114) (Zheng, et al., 2015). JQ1, a well-known BET inhibitor, was reported to block PCa cell growth (Chen and Song, 2016; Coleman, et al., 2019). Thus, JQ1 was used in the present study to further screen the hub SE-associated DEGs.

FKBP5 and TACC3 were still the key players deactivated by JQ1, while they were upregulated in mCRPC. Thus, we confirmed that FKBP5 and TACC3 might be the key candidates regulated by SEs in the pathogenic process of mCRPC. The TF regulatory network of mCRPC, the AR/FKBP5 axis and the ARNT/TACC3 axis were the focuses of our study. Consistent with a previous report (Mulholland, et al., 2011), AR was associated with FKBP5 in CRPC. Moreover, ARNT is a TF of basic helix-loop-helix/per-arnt-sim (PAS) family members, and predominantly heterodimerizes with the aryl hydrocarbon receptor (AHR) or hypoxia-inducible factor-1 alpha (HIF-1α), which has been linked to PCa angiogenesis (Fritz, et al., 2008). TACC3, which is involved in the pathogenesis of several cancers (Qie, et al., 2020), was markedly upregulated in CRPC. The ARNT/TACC3 complex is active in a hypoxic environment (Guo, et al., 2013). Our data suggest that CRPC progression may be regulated by the ARNT/TACC3 axis.

Single-cell analysis revealed that FKBP5 is mainly expressed in PCa cells of CRPC tissues. Therefore, FKBP5 might be a crucial SE-mediated gene in mCRPC. FKBP5, an androgen-inducible gene, physically interacts with AR (Zheng, et al., 2015). Recent studies have identified widespread activation of FKBP5 transcription in PCa cells by AR via distal intronic enhancers (Zheng, et al., 2015), which is related to chemoresistance in cancer (Li, et al., 2019). By contrast, little attention has been paid to the effects on ARNT/TACC3 axis in prostate cancer. Our previous study has demonstrated that CCR8-ARNT increased lactate production, and promoted aerobic glycolysis, which was related to poor outcomes of patients with advanced PCa (Chen G et al., 2020; Chen X et al., 2020). What’s more, recent studies showed that ARNT was related to tumor heterogeneity (Watkins, et al., 2020) and resistance of enzalutamide (Zhang, et al., 2022). ARNT, also known as hypoxia-inducible factor-1β (HIF-1β), has been recognized as an oncoprotein that promotes tumor growth in response to hypoxia (Harris, 2002), including multiple myeloma (Hassen, et al., 2015; Huang, et al., 2021), lymphoid cancer (Gardella, et al., 2016) and melanoma (Leick, et al., 2019). ARNT PAS-B domain was reported to interact directly with TACC3, which is a necessary step for transcriptional responses to hypoxia (Partch and Gardner, 2011). TACC3 gene is a centrosomal protein that is, involved in mitotic spindle assembly and chromosome segregation. Recent studies have shown that TACC3 is over-expressed in prostate cancer, and is associated with tumor progression and poor prognosis. Overexpression of TACC3 in prostate cancer cells has been shown to promote cell proliferation and migration. Therefore, it is reasonable to speculate that AR/FKBP5 axis and ARNT/TACC3 axis might play important roles in the progression of mCRPC.

## Conclusion

Our study provides valuable insights into the role of SEs in mCRPC development and suggests potential clinical applications for SEs.

## Data Availability

The datasets presented in this study can be found in online repositories. The names of the repository/repositories and accession number(s) can be found in the article/Supplementary Material.
